# Relationship between Urinary Pesticide Residue Levels and Neurotoxic Symptoms among Women on Farms in the Western Cape, South Africa

**DOI:** 10.3390/ijerph120606281

**Published:** 2015-06-02

**Authors:** Portia M. Motsoeneng, Mohamed A. Dalvie

**Affiliations:** Centre for Environmental and Occupational Health Research, School of Public Health and Family Medicine, University of Cape Town, Anzio Road, Observatory, Cape Town 7935, South Africa; E-Mail: mamonyowe.portia.64@gmail.com

**Keywords:** neurotoxicity, organophosphates, pyrethroid, pesticides, neurotoxic symptoms, Q16, female farm workers

## Abstract

*Background*: This cross-sectional study aimed to investigate the relationship between urinary pesticide residue levels and neurotoxic symptoms amongst women working on Western Cape farms in South Africa. *Method:* A total of 211 women were recruited from farms (*n* = 121) and neighbouring towns (*n* = 90). Participant assessment was via a Q16 questionnaire, reporting on pesticide exposures and measurement of urinary OP metabolite concentrations of dialkyl phosphates (DAP) and chlorpyriphos, 3,5,6-trichloropyridinol (TCPY) and of pyrethroid (PYR) metabolite concentrations (3- phenoxybenzoic acid (3PBA), 4-fluoro-3-phenoxybenzoic acid (4F3PBA), *cis*-2,2-dibromovinyl-2,2-dimethylcyclopropane-1-carboxylic acid (DBCA), and the *cis*- and *trans* isomers of 2,2-dichlorovinyl-2,2-dimethylcyclopropane-1-carboxylic acid. *Results:* Median urinary pesticide metabolites were slightly (6%–49%) elevated in the farm group compared to the town group, with 2 metabolites significantly higher and some lower in the farm group. The prevalence of all Q16 symptoms was higher amongst farm women compared to town women. Three Q16 symptoms (problems with buttoning, reading and notes) were significantly positively associated with three pyrethroid metabolites (*cis*- and *trans*-DCCA and DBCA), although associations may due to chance as multiple comparisons were made. The strongest association for a pyrethroid metabolite was between problems with buttoning and DBCA (odds ratio (OR) = 8.93, 95% confidence interval (CI):1.71–46.5. There was no association between Q16 symptoms and OP metabolites. *Conclusions*: Women farm residents and rural women from neighbouring towns in the Western Cape are exposed to OP and PYR pesticides. The study did not provide strong evidence that pesticides are associated with neurotoxic symptoms but associations found could be explored further.

## 1. Introduction

Organophosphate and pyrethroids insecticides, commonly used in agriculture have been associated with neurological deficits [1]. Neurological effects from exposure to or poisoning from to OP pesticides include problems with memory, sleeping, numbness, dizziness, weakness, confusion, depression, personality changes, thinking, concentration and language disabilities [2]. The neurotoxic effects of pesticide exposure can be summarised into both acute and chronic health effects. Acute neurotoxic effects are well studied and it is said to be caused by the inhibition of the enzyme acetylcholinesterase (AChE) causing changes in central nervous system function [3,4]. However, there remains conflicting information about the severity of chronic neurotoxic effects of pesticide exposure [2].

There are numerous epidemiological studies in the literature that have investigated the neurological effects of OP pesticides. Recently Ross *et al*. [2] conducted a systematic review on neurobehavioral problems associated with low-level exposure to OP pesticides for the period 1960–10th February 2012. The review found an overall significant relationship between low level OP exposure and cognitive functioning (language, general knowledge, attention psychomotor speed and memory). The review also showed that neurobehavioral health problems due to pesticides develop from prolonged exposure. Duration of OP exposure that can result in neurotoxicity ranged from 2 years to over 20 years. The review concluded that there was still uncertainty on the association between long term pesticide exposure and some neurobehavioral effects [2]. Most of these studies were conducted on men and women with no gender differences reported. Women are increasingly exposed to pesticides in agriculture [5].

There is limited evidence from two studies in the literature that have investigated neurotoxic effects of OP pesticides only on women and no studies investating PYR neurotoxicity [6,7]. Urinary concentration levels of pesticide metabolites such as the six dialkyl phosphate (DAP) metabolites of organophosphate pesticides, 3,5,6-trichloropyridinol (TCPY) which is a specific metabolite of chlorpyrifos and metabolites of pyrethroid pesticides have been shown to be higher in farm workers compared to the general population [8,9]. Previous studies investigating the association between urinary levels of pesticide metabolites and neurological health [10,11,12,13] were conducted in children and adolescents and not on adults. To our knowledge there is no previous study which has investigated the association between urinary levels of pesticide metabolites and neurotoxicity in adults.

South Africa is the highest user pesticides in sub-Saharan Africa and the Western Cape is an important agricultural area in the country [14,15]. Pesticide residues have been detected in environmental samples with high levels in farm workers [5,16,17]. One study has been conducted investigating neurological disorders due to agricultural pesticides amongst farm workers in the Western Cape and this study did not provide evidence of neurotoxicity due to OP exposure [18].

No previous studies have been conducted investigating the relationship between pesticide residues levels in biological samples and neuroxicity in South Africa. Female farm workers in South Africa are increasingly exposed to pesticides [19]. The data presented in this paper is part of a bigger study investigating neurotoxic, respiratory health and reproductive health effects of pesticide exposure among women living/working on farms in the Western Cape, South Africa. The aim of this study was to investigate the effect of occupational and environmental pesticide exposure on neurotoxic outcomes measured by means of the Q16 questionnaire.

## 2. Methods and Materials

### 2.1. Study Design, Population and Sampling

A cross-sectional study of women farm workers and residents and women living in towns neighbouring the farms, in the Western Cape region of South Africa was conducted during the period 24 October to 3 December 2009. The Women on Farms Project (WFP), a rural women’s rights non- governmental organisation, assisted with the recruitment of participants. About 100 women living on farms were targeted from the five most accessible agricultural areas representative of the Western Cape and 100 women from neighbouring towns that were about 5 to 10 km away from agricultural areas. The only inclusion criterion for women from these areas was age (*i.e*., above 18 years and below 70 years).

The study areas included Stellenbosch, Ceres, Paarl, Grabouw and Worcester. These are intensive crop farming areas producing table and wine grape and deciduous fruit (apples, peaches, prunes, pears). OP pesticides such as chlorpyrifos, methamidophos, azinphos methyl, monocrotophos, terbufos, parathion and fenamiphos and pyrethroids such as deltamethrin, permethrin, cypermethrin and cyfluthin are commonly used on these farms. Farm workers and residents were selected from the 5–10 most accessible and representative farms in each area and town women from the most accessible and representative houses in each area. One adult female participant per household was selected.

**Figure 1:**
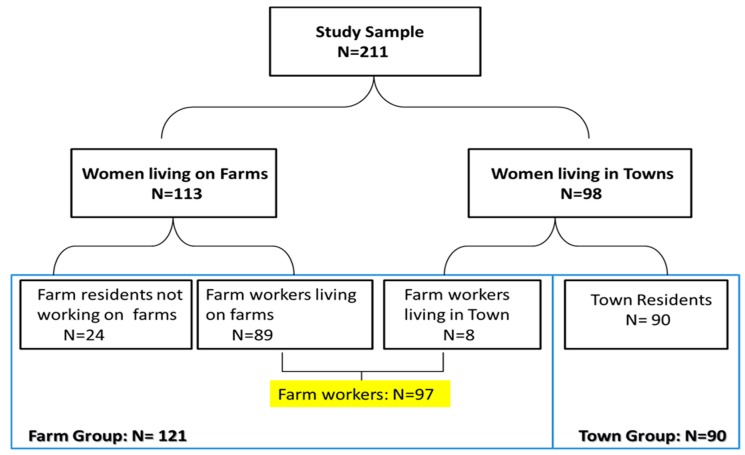
Diagram of sampling and participation in the study.

A total of 211 women were recruited into the study including 113 women currently living on a farm (including 89 farm workers and 24 farm residents not working on the farm) and 98 residents in neighbouring towns. Eight of the town residents actually worked on farms and were therefore classified as farm workers which increased the number of farm workers in the study to 97 farm workers (89 women living in farms and 8 not living in farms). The additional 24 women residing but not working on farms were included with the farm workers in the “farm group” (*n* = 121) as the results of sub-analysis showed they had similar results to that of farm workers. The remaining 90 women who neither lived nor worked on a farm are referred to as the “town group” (Figure 1).” The study was approved by the University of Cape Town’s (UCT) Research Ethics Committee (Reference 393/2009). Informed consent was obtained from participants prior to the interview.

### 2.2. Questionnaire

The questionnaire had sections on socio-demographic information (age, schooling, home language, income, employment); residential history (farm or town); pesticide household pesticide exposure; occupational and environmental pesticide exposure (being an applicator, re-entry pesticide exposure, pesticide drift, distance of residence to spraying and other exposures to agricultural spraying), job history (farm worker, non-farm worker, number of years in a job, job title), lifestyle factors (smoking, drug usage and alcohol consumption), pesticide poisoning and the Q16 questionnaire that has been shown to be effective in detecting differences in neurotoxic symptoms in groups of workers with varying exposures to organic solvents and has been used in a number of studies that investigated self-reported neurotoxic symptoms among workers who are exposed to toxic substances including pesticides [20,21,22]. The Q16 questionnaire which consists of 16 questions, with yes/no responses to symptoms associated with neurotoxicity. The instrument has been criticised for lacking sensitivity and specificity [23]. The study interviews were administered in the participants preferred language and the questionnaire was translated into Afrikaans and Xhosa and then back translated into English. Fieldwork was done on the WFP premises.

### 2.3. Urinary Pesticide Metabolites Determination

Urine samples were collected in 50 mL plastic containers on the same day as when the other testing was conducted. The period of testing for all participants was during the pesticide spraying season. Participants were instructed to take precautions not to contaminate samples by not removing contaminated clothing, making sure that they wash their hands before handling urine containers, not touching the inside of containers and closing the containers immediately after producing the sample. The samples were then kept on dry ice, and stored at −20 °C before being sent for analysis at the National Institute for Occupational Health (NIOH) laboratory in Johannesburg, South Africa. The urine samples were analysed for the organophosphate pesticide metabolites, dialkyl phosphates, the chlorpyrifos specific metabolite, TCPY and pyrethroid metabolites. The reasons for the choice of these pesticides include the fact that they had been associated with neurotoxicity, are commonly used on farms in these areas and the fact that the analytical methods were available at the laboratory.

Briefly, after allowing the samples to thaw at room temperature, 2 mL of urine was pipetted into screw top vials, which already contained approximately 2 g of sodium chloride. The samples were acidified and extracted. The extraction was repeated, and the two extracts were combined and dried. The dry residue was suspended in acetonitrile (500 µL). Derivatization was performed by adding pentafluorobenzyl bromide (50 µL). After cooling at room temperature the samples were reconstituted and transferred to gas chromatography ready for analysis.

Analysis was performed on an HP 6890 GC (Agilent Technologies, Santa Clara, CA, USA). For calibration a multi-component stock solution of all six dialkyl phosphate (20 µmol) metabolites, TCPY and PYR metabolites were used. For quality assurance, we used spike pooled urine at a concentration 2000 nmol/L for each of the dialkyl phosphate metabolites, TCPY and PYR metabolites.

The following metabolites were measured: OP metabolites (according to the methods by Hardt *et al*.) [24] including dimethyl phosphate (DMP), diethyl phosphate (DEP), dimethyl thiophosphate (DMTP), dimethyl dithiophosphate (DMDTP), diethyl thiophosphate (DETP), diethyl dithiophosphate (DEDTP); and 3,5,6-trichloropyridinol (TCPY), the specific chlorpyrifos metabolite [25] and the five PYR metabolites 3-phenoxybenzoic acid (3PBA), 4-fluoro-3-phenoxybenzoic acid (4F3PBA), *cis*-2,2-dibromovinyl-2,2-dimethylcyclopropane-1-carboxylic acid (DBCA), and *cis*- and trans isomers of 2,2-dichlorovinyl-2,2- dimethylcyclopropane-1-carboxylic acid (*cis*- and *trans*-DCCA) (according the methods of Arrebola *et al.* [26].

The limit of detection (LOD) for all analyses were determined and values too low to be quantified were assigned a value equivalent to the LOD/$\sqrt{2}$ (inverse of square root of 2). The limit of detection (LOD) for the pesticide metabolites were 0.5 μg/L for TCPY ; 1 μg/L for DMP; and 0.05 μg/L for DMTP, DMDTP, DEP, DETP, DEDTP, *cis*-DCCA, *trans*-DCCA, DBCA, 4F3PBA and 2PBA (*n* < LOD = 8, 1, 1.for TCPY, DAP and PYR respectively). There were eight, 16 and 11 insufficient urine samples for TCPY, DAP and PYR analysis respectively.

Results were adjusted for urinary creatinine to take account of hydration. Urine samples with creatinine concentrations within and outside the WHO recommended creatinine concentration range of 0.3 × 106 µg/L–3.0 × 106 µg/L were distinguished and taken into account during analysis. Those outside the WHO range are not presented (*n* = 18).

### 2.4. Variables

The outcome variables included the dichotomous (Yes, No) Q16 questions, a continuous Q16 score variable which was calculated as the sum of positive responses (positive responses coded as 1 and negative responses as 0) to Q16 questions. The Q16 score was also dichotomised at the median and 75th percentile. The exposure variables included the dichotomous self-reported history of living or working on farms (Yes, No), farm group/town group, and born on a farm as well as the urinary pesticide metabolite levels which were analysed as continuous variables.

### 2.5. Statistical Analysis

The selected software for analysis was Stata Release 11 (StataCorp LP, College Station, TX, USA). Since all continuous variables were not normally distributed, median and interquartile ranges were used to summarise these variables. After conducting univariate and bivariate analysis, multiple logistic regression analyses were used to test for associations between dichotomous outcomes and exposure variables while controlling for confounding and linear regression was used for the Q16 score. Confounders were selected on an *a priori* basis, according to biological plausibility, or based on their association with outcomes in bivariate testing if *p* < 0.1. Age, education, household income were selected *a priori* and drugs, alcohol usage, current smoking, language and previous poisoning were selected based on bivariate testing. Exposure variables were then added separately to all the different outcomes adjusting for these covariates.

To test for effect modification, interaction variables were created between exposure variables and potential effect modifiers (smoking, years of schooling and being born on a farm). These were the products between each exposure variable and a suspected effect modifier. For all the outcomes, an interaction term between the variable and the exposure variable of interest was included in the model. If this interaction term was significant (*p* < 0.05), the variable would be an effect modifier. None of the interaction terms were significant so all were not retained in the models.

## 3. Results

### 3.1. Participants

Two hundred and eleven women were recruited into the study with 20% (*n* = 42) coming from Ceres, 18% (*n* = 38) from Grabouw, 19% (*n* = 39) from Paarl, 22% (*n* = 47) from Stellenbosch and 21% (*n* = 45) from Worcester. Twenty-five (28%) of the women in the town group previously lived on farms. Among all the studied participants only two (2%) of the farm workers reported that they were applicators.

### 3.2. Demographic Information, Socio-Economic Status, Lifestyle Factors and Self-Reported Pesticide Exposure

In both groups, Afrikaans was the most spoken language (>87%) and less than 1% of the total studied population spoke English (Table 1). The median age in the town group was higher (40.5 years) than in the farm group (33 years) due to the fact that 25% of the town group was older than 50 years (excluding women aged higher than 50 years from the analysis did not change the results in the study). The number of women who attended school was not different in the two groups with over 96% of the participants in both groups who had attended school. The number of women who had matriculated was significantly more in the farm group although only 2% overall matriculated in both groups. Median household income was significantly higher in the town group. Unemployment was statistically significantly higher in the town group compared to the farm group (71% *vs.* 17% respectively). The occupations of town women included domestic worker (*n* = 3), beauty consultant (*n* = 1), administrator (*n* = 4), factory worker (*n* = 8), police woman (*n* = 1), community worker (*n* = 1), taxi driver (*n* = 1), self-employed (*n* = 5) and there was also a pensioner.

Alcohol consumption and smoking was more prevalent in the farm group. Household pesticide usage was prevalent in both groups, although slightly higher in the farm group. Household pesticide exposures were higher in the farm group including 10 (8%) who use empty containers. 

**Table 1:** Demographic information, socio-economic status, living and working history and lifestyle factors of participants in the study.

Characteristic	Farm Group (*n* = 121)	Town Group (*n* = 90)	Total (*n* = 211)
**Demographic Characteristics:**			
Age (years; Median, IQR)	33.0 (27.0–40.0)	40.5 (31.0–49.0)	37.0 (28.3–45.0)
Weight (Kg; *n* = 207; Median, IQR)	61.0 (51.0–72.1)	70.0 (58.3–81.1)	65.0 (54.0–75.1)
Home language n (%) English Afrikaans IsiXhosa	0 (0%) 119 (98%) 2 (2%) *	1 (1.1%) 79 (88%) 10 (11%)	1 (0.5%) 198 (94%) 12 (6%)
Level of Education: n (%) No schooling Matriculated	4 (3%) 1 (1%) *	4 (4%) 3 (3%)	8 (4%) 4 (2%)
Length of stay in current residence (years; Median, IQR)	15.0 (8–24)	21.5 (12–41)	17 (9–29)
Born on a farm: n (%)	83 (69)	13 (14)	96 (46)
History of ever living or working on farms	121 (100) *	26 (29)	147 (70)
**Socioeconomic Status**			
Unemployment: n (%)	20 (17)	65 (71)	85(40)
Household income/month ($US) (Median, IQR)	270.0 (188–500) *	378.7 (221–744)	324.0 (199–600)
**Lifestyle Factors n (%)**			
Current cigarette smoker	69 (57)	36 (40)	105 (50)
Current alcohol consumption	79 (65)	39 (43)	118 (56)
Use drugs	0 (0)	2 (2)	2 (0.01)

Abbreviations: IQR—Inter quartile range, Kg—kilograms, $US—United States dollar, n—number, %—percentage. Current cigarette smoker: having smoked at least 20 packs of cigarettes or 30 grams of tobacco in a lifetime or at least one cigarette per day for one year AND having smoked tobacco in the last month or more. * *p* ≤ 0.05 Statistical Tests: *t*-test (for normally distributed data) or Wilcoxon rank sum test (for data not normally distributed) was used for one dichotomous and one continuous variable, and Chi-square testing for 2 dichotomous variables.

**Table 2:** Household pesticide exposure, pesticide poisoning and agricultural pesticide exposure of participants.

Pesticide Exposure Household Pesticide Exposure	Farm Group N (%)	Town Group N (%)	Total N (%)
Use pesticides at home	67 (55)	56 (62)	123 (58)
Member of the family works as a pesticide applicator	36 (30)	1 (1)	37 (18)
Pesticide contaminated clothing washed at home	58 (48)	1 (1.1)	59 (28)
Clothing washed with rest of washing	39 (32)	0 (0.0)	39 (18)
Use of empty pesticide containers at home for drinking	10 (8)	0 (0.0)	10 (5)
**Pesticide Poisoning**			
Pesticide poisoning confirmed by a doctor	6 (5)	1 (1)	7 (3)
**Farm worker status** (*n* = 208)			
Permanent Seasonal	53 (45) * 40 (34) *	0 (0) 4 (4)	53 (25) 44 (21)
**Re-entry into sprayed fields** Delayed re-entry ^a^ Immediate re-entry ^b^	33 (27) * 81 (67) *	1 (1) 0 (0)	34 (16) 81 (38)

Abbreviations: N number, % percentage; * *p* ≤ 0.05; ^a^ re-entry into field 1 to 7 days after pesticide spraying; ^b^ re-entry into field on the same day after pesticide spraying.

As expected, past pesticide poisoning events diagnosed by a doctor were more prevalent in the farm group but low in both groups. On spraying days, about two thirds (67%) of the farm dwellers reported that they re-entered the field on the same day after pesticide spraying. Workers were employed for an average of five years on the farms and about a third of farm women were seasonal farm workers (Table 2). 64.1% of the farm group reported that pesticides were last applied in the last 2 days on the farm, 30.7% in the last 3–7 days and the rest between 14–33 days.

### 3.3. Urinary Pesticide Metabolite Results

Table 3 gives a summary of the urinary pesticides metabolites measured among the study participants. A total of 186 urine samples were collected from the participants of whom 18 had a creatinine concentration which was outside the WHO recommended range. For seven (4%) of the participants, the collected urine sample were not enough for measuring TCPY, for the dialkyl phosphates 15 (8%) and for pyrethroid 10 (5%). Most of the urinary organophosphate and pyrethroid metabolites were not significantly different between the two groups with only TCPY and *trans*-DCCA levels significantly higher in the farm group.

**Table 3:** Pesticide residues levels among rural female workers.

Pesticide Metabolites	Farm Group	Town Group	Total
Median (IQR)			
Corrected for Creatinine (µg/g Creatinine)			
**Organophosphate Metabolites *n* = 101 *n* = 77 *n* = 178**			
∑DAP	141.42 (37.4–249.83)	132 (45.64–204.45)	133.59 (41.86–229.09)
DMP	32.91 (13.50–55.75)	26.19 (14.33–52.36)	29.63 (14.06–53.22)
DMTP	13.41 (3.05–62.45)	36.44 (6.11–71.85)	21.87 (4.03–65.85)
DMDTP	5.70 (0.83–51.51)	9.57 (0.87–66.22)	6.87 (0.85–61.77)
DEP	5.01 (1.37–12.90)	4.13 (0.59–9.47)	4.27 (1.08–10.04)
DETP	3.70 (1.15–26.98)	3.94 (1.35–26.18)	3.87 (1.20–26.98)
DEDTP	1.99 (0.55–5.10)	1.70 (0.60–8.02)	1.89 (0.58–6.44)
**Chlorpyrifos Metabolite *n* = 104 *n* = 82 *n* = 186**			
TCPY	6.15 (3.50–10.64) *	4.14 (2.70–7.57)	5.16 (2.84–9.24)
**Pyrethroid Metabolites *n* = 101 *n* = 82 *n* = 183**			
∑Pyrethroids	6.60 (3.61–9.96)	5.26 (2.74–8.42)	6.01 (3.24–9.67)
*cis*-DCCA	0.71 (0.27–1.28)	0.56 (0.23–1.13)	0.62 (0.26–1.24)
*trans*-DCCA	0.85 (0.47–1.29) *	0.59 (0.28–1.02)	0.70 (0.37–1.22)
DBCA	0.31 (0.05–0.63)	0.30 (0.04–0.60)	0.30 (0.04–0.62)
4F3PBA	0.73 (0.31–1.32)	0.70 (0.33–1.30)	0.73 (0.32–1.32)
3PBA	3.61 (2.11–6.25)	3.34 (2.27–5.92)	3.40 (2.18–6.00)

* *p* ≤ 0.05; TCPY: 3,5,6-trichloropyridinol; DAP: sum of the 6 dialkyl phosphate metabolites; DMP: dimethyl phosphate; DMTP: dimethyl thiophosphate; DMDTP: dimethyl dithiophosphate; DEP: diethyl phosphate; DETP: diethyl thiophosphate; DEDTP: diethyl dithiophosphate; Pyrethroids: sum of the 5 pyrethroid metabolites; *cis*-DCCA: *cis*-2,2-dichlorovinyl-2,2-dimethylcyclopropane-1-carboxylic acid; *trans*-DCCA: *trans*-2,2-dichlorovinyl-2,2-dimethylcyclopropane-1-carboxylic acid; DBCA: *cis*-2,2-dibromovinyl-2,2-dimethylcyclopropane-1-carboxylic acid; 4F3PBA: 4-fluoro-3-phenoxybenzoic acid; 3PBA: 3-phenoxybenzoic acid; Values below LOD were substituted by LOD divided by square root of 2; ∑: total sum.

**Table 4:** Responses to Q16.

Neurotoxic Symptom	Farm Group N = 121 (57)	Town Group N = 90 (43)	Total N = 211 (100)
Are you abnormally tired? (tired)	81 (77) *	37 (41)	118 (56)
Do you have palpitations of the heart when you do not exert yourself? (heart palpitations)	60 (50) *	26 (29)	86 (41)
Do you often have painful tingling in some part of your body? (tingling)	55 (46) *	24 (27)	79 (37)
Do you often feel irritated without any particular reason? (irritated)	59 (49) *	22 (24)	81 (38)
Do you often feel depressed without any particular reason? (depressed)	62 (51) *	30 (33)	92 (44)
Do you often have problems concentrating? (poor concentration)	34 (28)	20 (22)	54 (26)
Do you have a short memory? (short memory)	59 (49) *	28 (31)	87 (41)
Do you often perspire without any particular reason? (perspire)	30 (25)	15 (17)	45 (21)
Do you have any problems with buttoning and unbuttoning? (button)	6 (5)	4 (4)	10 (5)
Do you generally find it hard to get the meaning from reading newspapers and books? (reading)	31 (26)	16 (18)	47 (22)
Have your relatives told you that you have a short memory? (family member)	32 (26)	18 (20)	50 (24)
Do you sometimes feel a heavy feeling on your chest? (chest)	48 (40) *	17 (19)	65 (31)
Do you often have to make notes about what you must remember? (notes)	36 (30) *	14 (16)	50 (24)
Do you often have to go back and check things you have done such as locking the door? (check door)	64 (53) *	26 (29)	90 (43)
Do you have a headache at least once a week? (headache)	105 (87) *	42 (47)	147 (70)
Do you think that you have less sex than most persons of your age? (less-sex)	53 (44)	35 (39)	88 (42)
Total Score (median, range) (q16 score)	7 (0–16) *	2.5 (0–15)	5 (0–16)
* *p* < 0.05 comparing Farm group to Town group			

* shows that there is a significant difference between the two groups.

### 3.4. Response to Q16 Questionnaire

Positive responses to individual items in the Q16 questionnaire were all more prevalent in the farm group with 10 (63%) items significantly higher in this group. The total score was therefore also significantly higher in the farm group (Table 4).

### 3.5. Multivariate Associations between Pesticides Exposure Indices and Q16 Questionnaire Items

Table 5, Table 6 and Table 7 below give details of the multivariate association between Q16 outcomes and pesticides exposure indices (farm group, history of ever living on a farm, born on a farm and pesticide residue levels) among the women who live on farms and neighbouring towns in the rural Western Cape areas. The prevalence of fifteen Q16 symptoms was higher in the farm group compared to the town group with 10 statistically significantly higher (tired, heart palpitations, tingling, irritated, depressed, short memory, chest, notes, check door and headache). Eight of the Q16 symptoms were significantly positively associated with history of ever living on a farm (tired, heart palpitation, irritated, tingling, poor concentration, short memory, perspire and chest). The sum of Q16 score was also significantly positively associated with farm group and history of living on a farm. Household pesticides was significantly associated with one Q16 symptom (button).

Three pyrethroids metabolites (*cis*-DCCA, *trans*-DCCA, DBCA) were significantly associated with Q16 symptoms. The strongest association was between DBCA and Q16 outcome “Button” (OR = 8.93, 95% CI: 1.71–46.5) (Table 7). “Button” and “Reading” were also significantly associated with trans DCCA and DBCA and “Notes” was significantly associated with and DBCA. There was no significant association between any Q16 symptom and any of the dialkyl phosphate and chlorpyrifos metabolites (Table 6). Excluding those previously poisoned from the analysis did not make a difference to the results.

## 4. Discussion

This study found that neurotoxic symptoms were significantly higher among women living and working on farms compared to those living in neighbouring towns which might only to a small extent be due to higher urinary levels of pesticide metabolites that were not substantially higher among farm women. The higher prevalence of neurotoxic symptoms among farm women might therefore be largely due to the fact that not all neurotoxic pesticides such as carbamates were measured in the study, residual confounding due to socio-economic status and lifestyle factors such as alcohol consumption and cigarette smoking or over reporting by farm women. The association between neurotoxic symptoms and farm exposure was found even when controlling for pesticide poisoning which have not previously been demonstrated with the Q16 questionnaire. Previous studies in Nicaragua and California have shown significantly higher positive symptoms responses in those that experienced poisoning compared to a non-poisoned group [27,28,29].

**Table 5:** Adjusted models for the association between residence/working on a farm, and neurotoxic symptoms among rural women in Western Cape.

Pesticide Exposure. Odds Ratio/Regression Coefficient (95% Confidence Interval)				
	History of ever Living and/or Workingon Farm	Born on Farm	Household Pesticides	Farm *vs.* Town Group
Q16 Outcomes				
Tired	**3.3 (1.46–7.36)**	0.95 (0.50–1.78)	0.61 (0.07–4.77)	**4.03 (2.07–7.86)**
Heart palpitations	**4.73 (1.98–11.31)**	1.29 (0.66–2.41)	0.44 (0.04–4.59)	**3.40 (1.70–6.78)**
Tingling	**4.72 (1.94–11.50)**	0.85 (0.44–1.62)	0.46 (0.04–5.07)	**3.81 (1.88–7.74)**
Irritated	**4.25 (1.82–9.95)**	0.77 (0.41–1.45)	1 (omitted)	**4.17 (2.08–8.36)**
Depression	1.89 (0.87–4.11)	0.91 (0.49–1.69)	0.40 (0.04–4.10)	**2.60 (1.38–4.88)**
Poor concentration	**4.15 (1.59–10.80)**	1.36 (0.67–2.77)	0.95 (0.09–9.95)	1.96 (0.93–4.12)
Short term memory	**2.94 (1.34–6.45)**	1.48 (0.78–2.79)	1.54 (0.20–11.73)	**3.03 (1.56–5.80)**
Perspire	**4.35 (1.42–13.31)**	1.05 (0.49–2.29)	0.76 (0.07–8.20)	1.69 (0.78–3.66)
Button	5.83 (0.56–60.74)	1.17 (0.28–4.94)	**10.35 (1.73–146.18)**	0.78 (0.19–3.25)
Reading	2.16 (0.79–5.86)	1.05 (0.51–2.32)	2.70 (0.34–21.37)	1.67 (0.76–3.65)
Fam mem	1.34 (0.54–3.36)	1.93 (0.88–4.25)	4.57 (0.58–35.88)	1.92 (0.88–4.16)
Chest	**5.21 (1.90–14.25)**	0.63 (0.31–1.29)	2.37 (0.30–18.91)	**3.84 (1.77–8.33)**
Notes	1.55 (0.64–3.77)	1.03 (0.49–2.19)	0.84 (0.08–9.05)	**2.47 (1.12–5.48)**
Check door	1.90 (0.85–4.23)	1.34 (0.71–2.54)	1.20 (0.16–9.30)	**3.10 (1.60–6.00)**
Headache	2.13 (0.91–5.00)	0.79 (0.40–1.56)	0.39 (0.05–3.03)	**9.41 (4.34–20.40)**
Less sex	1.70 (0.78–3.73)	0.71 (0.38–1.32)	0.49 (0.05–5.02)	1.29 (0.70–2.40)
Q16 score	**2.69 (1.71–10.14)**	2.10 (0.72–6.10)	0.07 (0.01–0.60)	**60.41 (6.96–524.51)**
Q16 score50	5.31 (2.22–12.69)	0.79 (0.42–1.51)	1.03 (0.13–7. 92)	5.27 (2.62–10.59)
Q16 score75	5.01 (1.76–14.25)	1.68 (0.77–3.54)	2.52 (0.32–19.72)	3.05 (1.39–6.87)

Confounder: Age, level of education, drugs, current smoking, alcohol consumption, household income, language, past pesticide poisoning.

**Table 6:** Adjusted models for the association between OP metabolites and Q16 outcomes among rural women in Western Cape.

Organophosphate Metabolites							
Dialkyl Phosphates. Odds Ratio Regression Coefficient (95% Confidence Interval)							Chlorpyrifos Metabolite
	DMP	DMTP	DMDTP	DEP	DETP	DEDTP	TCPY
Q16 outcomes							
Tired	0.998 (0.985–1.009)	1.001 (0.996–1.005)	0.998 (0.995–1.005)	1.006 (0.995–1.022)	0.995 (0.985–1.005)	1.004 (0.993–1.015)	1.005 (0.992–1.020)
Heart palpitations	0.990 (0.977–1.002)	0.999 (0.995–1.009)	1.002 (0.998–1.006)	1.003 (0.988–1.019)	0.995 (0.984–1.005)	0.997 (0.987–1.008)	1.007 (0.989–1.026)
Tingling	1.003 (0.989–1.009)	0.999 (0.995–1.003)	0.999 (0.995–1.004)	1.002 (0.978–1.017)	0.995 (0.984–1.006)	1.000 (0.989–1.011)	0.998 (0.988–1.007)
Irritated	0.997 (0.985–1.008)	1.001 (0.997–1.005)	1.000 (0.996–1.005)	1.002 (0.986–1.016)	0.993 (0.983–1.005)	0.995 (0.985–1.007)	1.021 (0.997–1.046)
Depression	1.002 (0.991–1.013)	1.000 (0.996–1.004)	0.999 (0.996–1.003)	0.999 (0.985–1.013)	0.994 (0.984–1.004)	0.998 (0.987–1.008)	1.006 (0.991–1.022)
Poor concentration	1.009 (0.9971–0.022)	1.000 (0.996–1.005)	0.996 (0.995–1.003)	0.994 (0.976–1.012)	0.998 (0.987–1.010)	0.999 (0.987–1.012)	0.929 (0.867–0.995)
Short term memory	1.005 (0.994–1.014)	1.000 (0.996–1.005)	1.000 (0.997–1.005)	0.996 (0.976–1.010)	0.989 (0.977–1.002)	0.994 (0.982–1.007)	1.000 (0.991–1.006)
Perspire	0.999 (0.985–1.014)	1.003 (0.998–1.007)	0.999 (0.994–1.004)	0.985 (0.959–1.011)	0.991 (0.976–1.001)	0.997 (0.981–1.012)	1.000 (0.990–1.009)
Button	1.010 (0.984–1.035)	1.003 (0.996–1.010)	0.994 (0.980–1.007)	0.972 (0.907–1.045)	1.000 (0.979–1.022)	0.966 (0.891–1.047)	1.000 (0.981–1.018)
Reading	0.997 (0.983–1.010)	1.005 (1.001–1.010)	0.999 (0.995–1.005)	0.986 (0.966–1.007)	0.998 (0.987–1.009)	0.995 (0.987–1.009)	0.993 (0.969–1.018)
Fam mem	0.997 (0.983–1.011)	0.996 (0.991–1.002)	1.002 (0.998–1.006)	0.995 (0.976–1.015)	0.995 (0.981–1.008)	1.003 (0.992–1.015)	0.991 (0.965–1.017)
Chest	0.993 (0.979–1.006)	1.001 (0.997–1.006)	1.004 (0.999–1.009)	0.994 (0.978–1.010)	0.993 (0.981–1.006)	0.996 (0.984–1.008)	0.998 (0.990–1.005)
Notes	1.009 (0.995–1.022)	1.004 (0.999–1.009)	0.998 (0.993–1.005)	0.991 (0.967–1.014)	0.996 (0.982–1.010)	1.002 (0.989–1.015)	0.999 (0.991–1.007)
Check door	1.006 (0.995–1.018)	0.999 (0.996–1.004)	1.997 (0.993–1.001)	0.992 (0.978–1.020)	0.999 (0.990–1.009)	1.000 (0.989–1.009)	0.990 (0.960–1.012)
Headache	0.995 (0.983–1.007)	1.001 (0.997–1.006)	0.999 (0.995–1.004)	0.999 (0.983–1.015)	1.000 (0.989–1.009)	1.002 (0.991–1.014)	1.011 (0.983–1.040)
Less sex	0.994 (0.982–1.005)	0.999 (0.995–1.007)	0.996 (0.995–1.000)	1.008 (0.993–1.024)	0.996 (0.985–1.006)	1.005 (0.994–1.015)	0.998 (0.990–1.005)
Q16 score	1.002 (0.984–1.020)	1.002 (0.996–1.006)	0.999 (0.993–1.006)	1.007 (0.981–1.032)	0.999 (0.985–1.010)	1.003 (0.986–1.021)	1.003 (0.981–1.026)
Q16 score50	1.000 (0.989–1.012)	1.001 (0.997–1.005)	0.999 (0.996–1.005)	0.995 (0.975–1.007)	0.991 (0.980–1.003)	0.998 (0.987–1.009)	0.998 (0.989–1.005)
Q16 score75	1.006 (0.992–1.019)	1.002 (0.997–1.007)	1.000 (0.995–1.005)	0.996 (0.971–1.010)	0.995 (0.982–1.008)	0.998 (0.985–1.012)	0.997 (0.981–1.01)

Confounder: Age, level of education, drugs, current smoking, alcohol consumption, household income, language, past pesticide poisoning.

**Table 7:** Adjusted models for the association between pyrethroid metabolites and Q16 outcomes among rural women in Western Cape.

Pesticide Exposure						
Pyrethroids. Odds Ratio Regression Coefficient (95% Confidence Interval)						
	*cis*-DCCA		*trans*-DCCA	DBCA	4F3PBA	3PBA
Neurotoxic outcomes						
Tired		1.22 (0.74–2.00)	1.44 (0.81–2.56)	1.91 (0.80–4.55)	1.16 (0.80–1.68)	1.00 (0.98–1.02)
Heart palpitations		1.03 (0.63–1.66)	1.17 (0.72–1.89)	1.14 (0.49–2.64)	0.92 (0.65–1.32)	1.00 (0.98–1.02)
Tingling		0.81 (0.488–1.34)	0.92 (0.56–1.54)	0.82 (0.34–1.95)	0.73 (0.46–1.14)	1.00 (0.98–1.02)
Irritated		1.02 (0.63–1.65)	1.18 (0.73–1.90)	1.34 (0.58–3.07)	0.94 (0.66–1.34)	1.00 (0.98–1.02)
Depression		1.05 (0.67–1.66)	1.10 (0.69–1.76)	1.54 (0.69–3.42)	0.96 (0.68–1.34)	1.00 (0.98–1.02)
Poor concentration		1.06 (0.63–1.78)	0.93 (0.55–1.59)	1.49 (0.61–3.65)	0.82 (0.52–1.28)	0.97 (0.91–1.03)
Short term memory		1.00 (0.61–1.62)	1.14 (0.70–1.85)	1.35 (0.58–3.13)	0.78 (0.51–1.18)	1.00 (0.98–1.02)
Perspire		1.00 (0.55–1.74)	1.11 (0.65–1.90)	1.22 (0.46–3.29)	0.72 (0.42–1.23)	1.01 (0.99–1.03)
**Button**		**3.03 (1.22–7.50**)	2.47 (0.94–6.45)	**8.93 (1.71–46.5)**	1.47 (0.85–2.54)	1.02 (0.99–1.05)
**Reading**		1.57 (0.92–2.67)	1.63 (0.94–2.83)	**2.95 (1.16–7.54)**	1.08 (0.74–1.57)	1.01 (0.99–1.03)
Fam mem		1.08 (0.63–1.87)	1.01 (0.59–1.73)	1.45 (0.56–3.78)	0.90 (0.56–1.45)	1.00 (0.97–1.03)
Chest		0.96 (0.57–1.60)	0.94 (0.57–1.57)	1.12 (0.46–2.76)	0.62 (0.38–1.04)	1.00 (0.98–1.02)
**Notes**		1.54 (0.88–2.71)	**1.82 (1.00–3.32)**	**2.82 (1.04–7.63)**	1.19 (0.81–1.75)	1.00 (0.97–1.02)
Check door		1.17 (0.74–1.86)	1.43 (0.85–2.39)	1.53 (0.68–3.48)	1.09 (0.77–1.53)	1.00 (0.98–1.02)
Headache		1.11 (0.66–1.85)	1.03 (0.60–1.77)	1.04 (0.43–2.52)	0.97 (0.67–1.39)	0.98 (0.96–1.01)
Less sex		0.85 (0.53–1.38)	0.88 (0.54–1.43)	0.66 (0.28–1.54)	0.77 (0.51–1.15)	0.99 (0.96–1.02)
Q16 score		1.32 (0.60–2.92)	1.35 (0.53–3.42)	1.46 (0.38–5.63)	0.93 (0.55–1.56)	0.98 (0.96–1.01)
Q16 score50		1.06 (0.66–1.71)	1.10 (0.68–1.79)	1.56 (0.68–3.59)	0.82 (0.56–1.20)	1.00 (0.98–1.02)
Q16 score75		1.12 (0.65–1.92)	1.29 (0.76–2.20)	2.06 (0.80–5.25)	0.87 (0.55–1.37)	1.01 (0.99–1.03)

Confounder: Age, level of education, drugs, current smoking, alcohol consumption, household income, language, past pesticide poisoning.

The study results showed no significant association between urinary metabolite levels of organophosphates, the most commonly used neurotoxic pesticides worldwide [8,30] and in South Africa and the Q16 outcomes. The median levels of DAP metabolites in this study (134 µg/g of creatinine) were lower than that measured in a previous study in the Western Cape among farm workers (1587.5 μg/g creatinine [19]. In this study median DAP levels were also at the low end of the spectrum when compared to those of Dutch farm workers in another setting (296.0 μg/g creatinine) [31]. The reason for no positive associations of DAP metabolites with Q16 outcomes could be therefore due to low levels of total organophosphate pesticide exposure of the female participants in this study. The low level OP exposure is probably due to the fact that only two of the farm workers reported that they were applicators. Another reason for the lack of association between OP metabolites could be due to the lack of specificity and sensitivity of the Q16 questionnaire [23] and that more sensitive neurotoxic tests are required to explore this association.

Significant associations between urinary PYR metabolites and neurotoxic symptoms could be due to chance as multiple comparisons were made for analyzing associations between urinary levels of pesticides and neurotoxic symptoms (213 comparisons made of which 2.3% (*n* = 5) were significant at the 5% level). *cis*- and *trans*-DCCA are metabolites for permethrin, cypermethrin and cyfluthrin that are commonly used on farms in the Western Cape crop farming; DBCA, is the metabolite of deltamethrin and 4F3PBA, a metabolite of cyfluthrin which are also both commonly used on Western Cape farms. 3PBA is a non-specific metabolite for common synthetic pyrethroids [32]. The median PYR metabolites measured in this study in both the farm and town groups (6.60 μg/g creatinine and 5.26 μg/g creatinine respectively) was higher than those measured in the general population in other countries such as in the Mexican study, MICASA [33] and the two USA population based studies NHANES data set 1999–2002 and CHAMACOS cohort with U.S. National Health and Nutrition Examination Survey data set 1999–2002.

We could not find another epidemiological study that investigated the relationship between pyrethroid levels and neurotoxic outcomes but, altered nerve functioning has been found in rats dosed with pyrethroid compounds through intra-cerebral dosing experiment [34].

The positive associations between PYR metabolites, *cis*-DCCA, *trans*-DCCA, DBCA and Q16 symptoms should be studied further using sensitive neurotoxic outcomes such as the World Health Organisation Neurobehavioral Core Test Battery and the Brief Symptom Inventory and vibration sense threshold testing. With most of the positive associations with the three PYR metabolites not significant, there is indication of the lack in statistical power in current study and a larger sample size would be required for future studies.

It is interesting that the levels of OP and PYR metabolites amongst women in the Town Group were also substantially higher than those in general populations [33]. This indicates that those residents who live in towns are also exposed to pesticides. The most likely pesticide exposures in rural towns include household pesticide and environmental exposure to agricultural pesticides.

A key limitation in this study is the cross‑sectional design; consequently it cannot be established with certainty if the associations are the result of a temporal relationship between pesticide exposure and outcomes. The short half-lives (<48 h) of the pesticides in the body [15] is particularly relevant here as exposures would be variable and one spot urine samples is not an ideal indicator of exposure. However, samples were taken in the spraying season with 64% of participants reporting that the last spray on the farm occurred less than 2 days ago and 95% less than 7 days ago. A longitudinal design whereby pesticide exposure especially urinary pesticide metabolites and neurotoxic outcomes are measured repeatedly over time would be more powerful. With respect to the comparison of Q16 symptoms between the farm group and town group, the healthy worker effect commonly observed in cross-sectional studies may have resulted in farm workers affected by pesticides to move to towns and thereby reducing the level of neurotoxicity in the farm group. However, the study results show Q16 symptoms were significantly higher in the farm group (Table 5) despite a possible health worker effect. Additionally, Q16 symptoms were significantly higher among women with a history of ever living and/or working on farm compared to those without such a history (Table 5). Furthermore sub-analyses excluding town women who had previously lived or worked on farm from the analyses did not change the results found.

Another important limitation in the study is the fact that age, income and employment status in the farm group and town group were different. These variables were not found to have strong associations with the Q16 symptoms in bivariate analysis and age and income were controlled for in multivariate analysis as they were included *apriori.* There might, however, have been residual confounding especially with income as the only indicator of socio-economic status. The most important limitations in the study was a lack of a sensitive outcomes, and the cross-sectional design which precludes the determination of the temporal effects and also a lack of statistical power due to a small sample size. With a larger study cohort study incorporating sensitive neurotoxic outcomes and multiple pesticide bio-monitoring measurements could have been conducted. The high prevalence of cigarette smoking and alcohol consumption among farm workers and residents is related to poverty and the previous use of alcohol as remuneration on farms.

## 5. Conclusions

This study found that urinary levels of DAP metabolites of rural women in the Western Cape to be lower than those in other settings, but PYR metabolites to be higher than those in other settings. The prevalence of all Q16 symptoms was higher amongst farm women compared to non-farm women. Three urinary pyrethroids metabolites (*cis*-DCCA, *trans*-DCCA, DBCA) were significantly positively associated neurotoxic symptoms adjusting for confounders, however, the associations may be due to chance. The results are suggestive and need further investigations in populations with a greater range of pesticide exposures and also in a bigger longitudinal study using more sensitive neurotoxic measures. The study results highlight the need to develop strategies to reduce pesticide exposure among women farm workers and residents.
